# Decrease in Facial Bone Density with Aging and Maintenance Effect of Calcium Maltobionate Ingestion in Japanese Adult Women: A Randomized, Double-Blind, Placebo-Controlled, Parallel-Group Trial

**DOI:** 10.3390/nu17020262

**Published:** 2025-01-12

**Authors:** Daiki Suehiro, Nami Ikeda, Kiyoto Hirooka, Akinori Ihara, Ken Fukami, Motoko Ohnishi

**Affiliations:** 1 San-ei Sucrochemical Co., Ltd., 24-5, Kitahama-machi, Chita-city 478-8503, Aichi, Japan; daiki-suehiro@sanei-toka.co.jp (D.S.); journal@sanei-toka.co.jp (K.F.); 2 Graduate School of Bioscience and Biotechnology, Chubu University, 1200, Matsumoto-cho, Kasugai-city 487-8501, Aichi, Japan; soneshow.7372@gmail.com; 3 Medical Foundation Co., Ltd., 1477-5, Shimizu-cho, Sunto-gun 411-0917, Shizuoka, Japan; ctmobileinc2024@gmail.com; 4 Ihara Machinaka Dental Clinic, 1-3-10, Haramachinaka, Numazu-city 410-0311, Shizuoka, Japan; ihara.machinaka.dc@gmail.com; 5 The College of Bioscience and Biotechnology, Chubu University, 1200, Matsumoto-cho, Kasugai-city 487-8501, Aichi, Japan

**Keywords:** facial bone density, calcium maltobionate, maltobionic acid, bone metabolism, randomized controlled trial

## Abstract

Background/Objectives: Facial bone density, including the jawbone, declines earlier than that of the lumbar spine and calcaneus. Calcium maltobionate is reported to mitigate bone resorption and maintain bone density of the lumbar spine in post-menopausal women, but its effects on facial bone density remain understudied. Therefore, this study compared variations in facial bone mineral density with variations in calcaneal bone mineral density and bone resorption markers among healthy women, examining differences between pre- and post-menopause and the effects of continuous calcium maltobionate intake. Methods: This randomized, double-blind, placebo-controlled, parallel-group trial involved 48 healthy Japanese women aged 30–69 years, divided into two groups. The test food group received tablets containing calcium maltobionate, while the placebo group received tablets containing a maltose and calcium carbonate mixture for 24 weeks. Calcaneal and facial bone densities were measured pre- and post-intervention in both groups. Results: Post-intervention calcaneal bone mineral density and bone resorption marker deoxypyridinoline (DPD) showed no statistical difference between groups in pre-menopausal women. However, in post-menopausal women, the test food group exhibited significantly higher calcaneal bone density and lower DPD levels compared with the placebo group. Facial bone mineral density increased significantly in the test food group compared with the placebo group in post-menopausal participants, with similar trends observed in pre-menopausal participants. Conclusions: Facial bone mineral density could serve as a useful indicator for monitoring bone health from middle age onward. Moreover, continuous calcium maltobionate intake appears to mitigate bone density decline in pre- and post-menopausal women, contributing to osteoporosis prevention (UMIN-CTR ID: 000046391).

## 1. Introduction

Bone serves as a calcium reservoir and a supporting tissue for the organism. In living organisms, the maintenance of mineral homeostasis and bone tissue metabolism involves repeated osteoclast-mediated bone resorption and osteoblast-mediated bone formation [1,2]. However, when this balance is disrupted, and the bone resorption rate exceeds bone formation, bone density decreases, thereby increasing the risk of osteoporosis [3,4]. Estrogen deficiency, particularly in post-menopausal women, plays a significant role in the development of osteoporosis by inducing osteoclast activation and decreasing bone mass [5,6]. Even before menopause, in the patient’s late 30s and 40s, hypoestrogenism progresses, resulting in subjective symptoms such as palpitations, dizziness, and abnormal sweating associated with disturbed autonomy [7,8]. This is the period when bone maintenance transitions to a gradual decline [9]. Research indicates that bone mineral density decreases markedly earlier in alveolar and jaw bones, with smaller trabecular bone areas than in the lumbar spine, femur, and calcaneus, which are primary measurement sites for osteoporosis diagnosis [10,11]. Moreover, bone mineral density rapidly decreases in the maxilla and mandible in both men and women after turning 40 [11].

Maltobionic acid is an indigestible disaccharide composed of gluconic acid linked to glucose by an α-1,4 glycosidic bond. It has the characteristic of stable mineral component dissolution in a wide range of pHs, from acidic to alkaline medium. In addition, its solubility is considerably greater than that of existing calcium compounds, such as calcium carbonate, calcium citrate, and calcium lactate, which are commonly used in calcium supplements [12]. Animal studies in rats have shown that maltobionic acid solubilizes minerals in the intestinal tract and increases mineral retention rates in the body, including calcium, magnesium, and iron [12,13]. Clinical trials have indicated that consuming calcium maltobionate promotes higher calcium absorption compared with calcium carbonate [14]. Furthermore, in vitro studies have demonstrated that maltobionic acid inhibits osteoclast differentiation, while in vivo studies using menopausal model mice have shown a significant decrease in bone resorption markers [15]. Clinical trials on maltobionate or calcium maltobionate intake in post-menopausal women have reported reductions in bone resorption markers, such as deoxypyridinoline (DPD) and type I collagen-cross-linked N-telopeptide [16], along with maintenance of lumbar spine bone density [17].

However, there is currently limited knowledge regarding the effects of maltobionic acid or calcium maltobionate on bone density in pre-menopausal women and the efficacy of their bone resorption inhibitory effects. Therefore, in this study, we aimed to compare variations in facial bone density, which reportedly decreases at an earlier stage, with variations in calcaneal bone density and bone resorption markers in healthy Japanese adult women. Furthermore, we aimed to examine differences between pre- and post-menopause and assess the effects of continuous calcium maltobionate intake.

## 2. Methods

### 2.1. Study Design and Participants

This randomized, double-blind, placebo-controlled, parallel-group trial recruited healthy Japanese women aged 30–69 years affiliated with Chubu University (Aichi, Japan). Detailed information about the study was provided to interested individuals during a research briefing session. Those who provided written informed consent completed a preliminary questionnaire and underwent a screening test by a dentist. Exclusion criteria were as follows: (a) a medical history of heart failure, myocardial infarction, or malignant tumors; (b) undergoing treatment for chronic diseases, including cardiac arrhythmia, osteoporosis, renal disorder, hepatic disorder, cerebrovascular disorder, diabetes mellitus, or rheumatism; (c) regular consumption of supplements affecting bone metabolism, such as calcium, magnesium, vitamin D, vitamin K, and isoflavones (including equol, genistein, and daidzein); (d) known allergy to the test food products; (e) a calcaneal young adult mean (YAM) of 70% or lower in bone mass measurements; (f) deemed unsuitable for participation by the dentist, including individuals with severe caries or alveolar pyorrhea.

The Chubu University Certified Review Board approved the study protocol on 24 December 2020 (approval no. 0200067-2). The study was conducted in accordance with the medical ethics outlined in the Declaration of Helsinki (2013) and the Ethical Guidelines for Medical and Health Research Involving Human Subjects. Written informed consent was obtained from all participants included in the study. This study was registered with the University Hospital Medical Information Network Clinical Trials Registry (UMIN-CTR ID: 000046391).

### 2.2. Sample Size

In a previous 24-week intervention study involving the continuous intake of calcium maltobionate or calcium carbonate, effect sizes were calculated based on differences in lumbar spine bone mineral density between the two groups (Hedges’ g: 1.16) [17]. With a significance level (α) of 0.05 and statistical power (1 − β) of 0.95, 42 participants (21 in each group) were required to achieve statistical significance. To account for potential dropouts and protocol compliance violations during the study period, the sample size was increased to 52 participants (26 in each group).

### 2.3. Selection, Randomization, and Blinding

The 52 eligible participants were divided into the test or placebo food group, with 26 participants in each group. Similar distributions of age, menopausal status, and YAM values of the right calcaneus were ensured across both groups. Randomization was conducted by the allocation manager using randMS (FileMaker, Inc., Cupertino, CA, USA) based on provided identification numbers. The allocation ratio for both groups was 1:1. Participants, outcome assessors, and other study personnel were blinded to group assignments throughout the study and were not involved in the allocation process.

### 2.4. Intervention Foods

**Figure 1:**
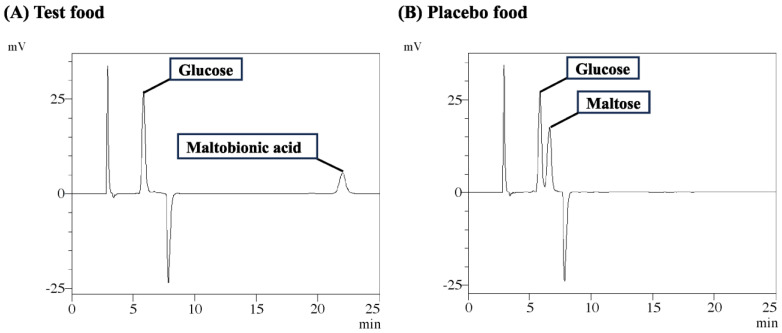
High-performance liquid chromatogram (HPLC) of test food and placebo food. An HPLC analyzing the amount of maltobionic acid in test foods and placebo foods is shown. The amount of maltobionic acid in test foods (tablets) was determined by an HPLC under the following analytical conditions: guard column, Asahipak NH2P-50G 4A, 4.6 × 10 mm (Resonac Corporation, Tokyo, Japan); column, Asahipak NH2P-50G 4A, 4.6 × 250 mm (Resonac Corporation, Tokyo, Japan); eluent system, acetonitrile/21 mM citric acid-21 mM disodium hydrogen phosphate (60/40); flow rate, 0.8 mL/min; column temp., 40 °C; detection, RI; injection vol., 10 µL.

The intervention involved tablets containing calcium maltobionate (San-ei Sucrochemical Co., Ltd., Chita, Japan), prepared by the action of glucose oxidase on maltose. Each tablet contained 1.100 g (1.045 g as maltobionic acid) per 5 g of tablets and was used as the test food. Placebo tablets had the same composition, except that calcium maltobionate was substituted with a mixture of maltose and calcium carbonate. The formulations and daily nutritional compositions of both the test and placebo foods are shown in Table 1. The chromatograms and analytical conditions for the test and placebo foods are shown in Figure 1.

Participants were instructed to consume two tablets daily (5 g) at their discretion. The intervention period lasted 24 weeks, during which participants were encouraged to maintain their usual lifestyle habits, including diet and exercise.

### 2.5. Nutritional Survey

Calcium, magnesium, phosphorus, potassium, vitamin D, and vitamin K are crucial for maintaining bone mineral density and regulating bone turnover [18,19,20,21,22]. These nutrients are expected to have similar efficacy as the test foods used in this study. To accurately assess the effectiveness of the test foods, participants’ diets were assessed before and after the intervention using a validated self-administered brief-type diet history questionnaire (BDHQ) [23,24]. The BDHQ is a questionnaire that investigates the consumption of 56 foods and beverages over the past month. Dietary intakes of energy and selected nutrients (calcium, magnesium, phosphorus, potassium, vitamin D, vitamin K, carbohydrates, lipids, and proteins) were estimated using computer-based algorithms.

### 2.6. Outcome Measures

#### 2.6.1. Calcaneal Bone Density

Calcaneal bone mineral density was assessed using an ultrasonic bone densitometry device (CM-200, Canon Lifecare Solutions Inc., Tokyo, Japan) through quantitative ultrasonography (QUS). Measurements were taken before intervention and after 24 weeks, with the right calcaneus designated as the measurement site, using ultrasonic transmission velocity, specifically the speed of sound (SOS), expressed as the YAM value. The YAM value shows the SOS of participants as a percentage, with an SOS mean of 1538 m/s defined as 100% for young adults.

#### 2.6.2. Facial Bone Density

During the screening examination, study participants received a mouthpiece (2.3 mm thick) made of vinyl ethylene acetate after obtaining an impression of the entire mandible, including the mandibular second molar, using a silicone impression material (FUSION II, GC Corporation, Tokyo, Japan). The mouthpiece was fitted into each participant’s mandible. Five sites were marked with 1.5 mm hemispherical gutta-percha points on both the labial and lingual sides: between the right second molar and right first molar (7-6), between the right first premolar and right canine (4-3), between the right and left central incisors (1-1), between the left canine and left first premolar (3-4), and between the left first molar and left second molar (6-7). For computed tomography (CT) imaging, the mouthpiece remained attached to the mandible, and a dental CT imaging device (KaVo 3D eXam, KaVo, Dental Systems, Biberach, Germany) was used to capture images from the mental to the orbital region in panoramic CT mode (70 kV, 6 mA, 17.8 s, caliber 23 cm, and height 17 cm) both before and 24 weeks after intervention.

**Figure 2:**
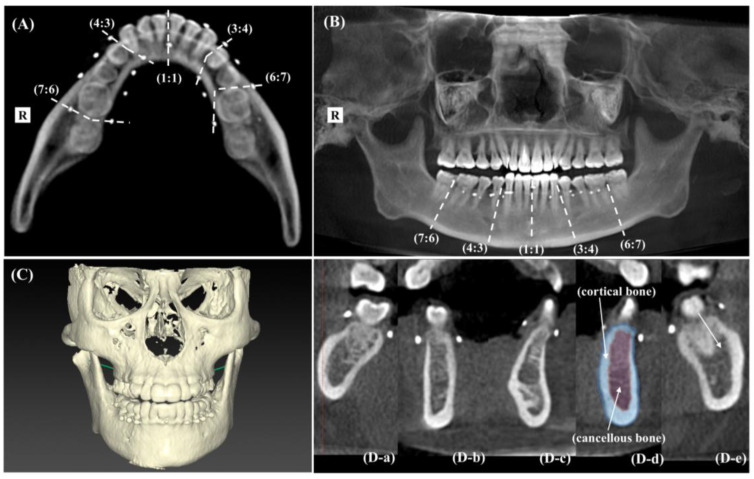
Sites of computed tomography (CT) and analytical procedures. (**A**) The location of CT imaging sites marked on the mandibular stent; (**B**) a CT image of the frontal face, including the region from the orbital area to the mental foramen; (**C**) the three-dimensional CT image of the frontal face, covering the region from the orbital area to the mental foramen. (**D**) Cross-sectional slice images at each site of the mandible: (**D-a**), the right second molar and right first molar (7-6); (**D-b**), the right first premolar and right canine (4-3); (**D-c**), the right and left central incisors (1-1); (**D-d**), the left canine and left first premolar (3-4); (**D-e**), the left first molar and left second molar (6-7).

Bone mineral density was assessed using CT imaging data from DTX Studio™ Implant (Nobel Biocare Inc., Gothenburg, Sweden). The X-ray absorbance factor, measured in Hounsfield units (HU), was calculated from the image analysis. HU was expressed as the relative value of the attenuation factor of each bone tissue, with water set as 0. The HU value was calculated using the following formula: HU value = (μt − μw)/μw × 1000, where μt represents the coefficient of attenuation of bone tissue, and μw represents the coefficient of attenuation of water. The analysis included (1) five points on cross-sectional slice images connecting the two points on the labial and lingual sides of the mandible with arbitrary points, (2) the periphery of the mental foramen, (3) the periphery of the supraorbital foramen, and (4) the periphery of the infraorbital foramen. The boundary division between the cortical bone plane and the cancellous bone (medullary cavity) plane was determined following Nakatsuchi et al.’s method [25], and HU values were calculated for both. The radiographic CT sites are shown in Figure 2.

For CT imaging, the mouthpiece remained securely attached to the mandible, and a dental CT imaging device (KaVo 3D eXam; KaVo, Dental Systems, Biberach, Germany) was used to capture images from the mental to the orbital region in panoramic CT mode (70 kV, 6 mA, 17.8 s, caliber 23 cm, and height 17 cm) both before and 24 weeks after the intervention. Bone mineral density was analyzed using CT imaging data from DTX Studio™ Implant (Nobel Biocare Inc., Gothenburg, Sweden). The X-ray absorbance factor in Hounsfield units was calculated using image analysis.

#### 2.6.3. Bone Resorption Marker

Approximately 10 mL of first-morning urine were collected the day before the intervention began and the day after the intervention ended. Urine samples were corrected for volume using urinary creatinine equivalents, and urinary DPD was measured as a bone resorption marker. Urinary DPD levels were quantified using an enzyme-linked immunosorbent assay (ELISA) kit (Osteolinks-DPD; SB Bioscience Co., Ltd., Osaka, Japan). Urinary creatinine levels were measured using the LabAssay™ Creatinine kit (FUJIFILM Wako Pure Chemical Corporation, Osaka, Japan).

### 2.7. Statistical Analysis

All outcomes are presented as the mean ± standard error (SE). Both within-group and between-group comparisons of results were conducted. Within-group comparisons were performed using paired the Student’s *t*-test or Welch’s *t*-test, taking into account data normality and equidispersiveness, to evaluate differences between pre-intervention and 24-week post-intervention measures. Between-group comparisons were conducted by comparing measurements and changes at each time point between the test and placebo food groups. Changes were calculated by subtracting pre-intervention values from 24-week post-intervention values. Pre-intervention measures and changes were determined using the Student’s *t*-test, while 24-week post-intervention measures were compared between groups using pre-intervention measurements as covariates. Post hoc analyses with repeated measures of ANCOVA were conducted to investigate interactions among the menopausal status (pre-menopausal and post-menopausal), placebo food group, and test food group. All statistical analyses were performed using two-tailed tests, with the significance level set at 5%. Analyses were conducted using Microsoft Excel for Microsoft Office 365 (Microsoft Japan, Tokyo, Japan) and BellCurve for Excel (Social Survey Research Information, Tokyo, Japan). Multiplicity adjustments at other time points or for other variables were not considered.

## 3. Results

### 3.1. Study Setup and Participant Demographics

**Figure 3:**
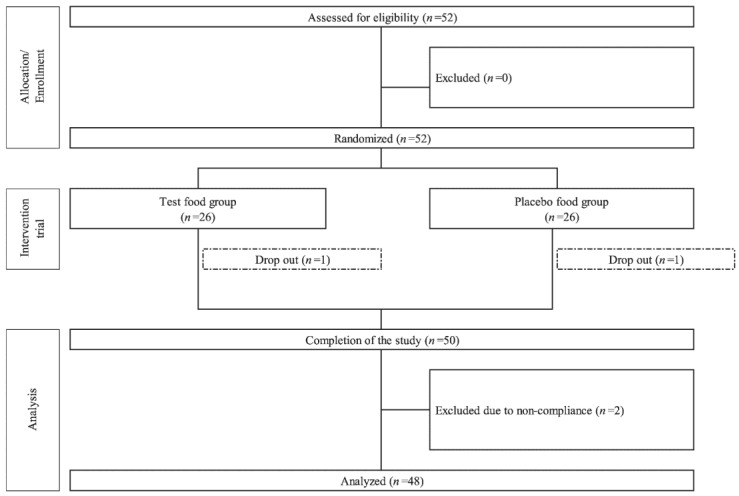
Flow chart for study participants’ follow-up. This was a randomized, double-blind, placebo-controlled, parallel-group trial.

Figure 3 shows the flowchart of study participants, and Table 2 provides the demographic characteristics of the 48 participants included in the analysis. Two dropouts and two non-compliant participants (with test or placebo food consumption rates of ≤80%) were excluded from the study. Therefore, the analysis was conducted as the “per protocol set”, which comprised 24 participants (average age of these 24 participants: 52.5 ± 1.4 years, that of 8 pre-menopausal women: 45.1 ± 2.0 years, and that of 16 post-menopausal women: 56.2 ± 1.0 years) in the test food group and 24 participants (average age of 24 participants: 54.0 ± 1.3 years, that of 8 pre-menopausal women: 48.3 ± 2.1 years, and that of 16 post-menopausal women; 56.9 ± 1.0 years) in the placebo food group. There were no significant differences in participant demographics between the groups. Participants were recruited between 1 October and 15 October 2021, and the study was conducted from 4 November 2021 to 14 June 2022.

### 3.2. Nutritional Survey

Table 3 presents the results of the BDHQ dietary survey. Mineral and vitamin intake (calcium, potassium, magnesium, phosphorus, vitamin D, and vitamin K) related to bone mineral density and metabolism did not significantly differ in either within-group or between-group comparisons across pre- and post-menopausal analysis populations.

### 3.3. Efficacy Assessment

#### 3.3.1. Calcaneal Bone Density

**Figure 4:**
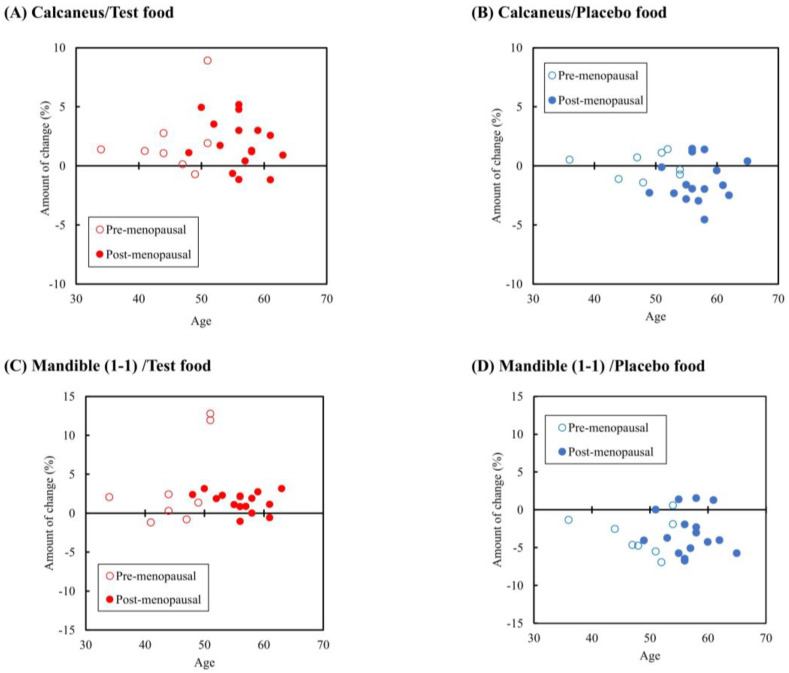
Age correlations for the amount of bone mineral density change in the calcaneus and mandible. The above figure shows the correlations between pre- and post-intervention changes in the calcaneal speed of sound (SOS) value and mandibular (1-1) Hounsfield unit (HU) value in relation to study participant age. (**A**) The correlation between the percentage change in the calcaneal SOS value and age in the test food group; (**B**) the correlation between the calcaneal SOS value and age in the placebo food group; (**C**) the correlation between the percentage change in the HU value of the mandible (1-1) and age in the test food group; (**D**) the correlation between the percentage change in the HU value of the mandible (1-1) and age in the placebo food group.

The calcaneal bone density results are shown in Table 4 and Figure 4A,B. When considering only post-menopausal participants, the test food group exhibited significantly higher levels of YAM values than the placebo food group at 24 weeks post-intervention. And, the test food group exhibited signifi-cantly higher level of both SOS and YAM value than the placebo food group in the change between pre-intervention and 24 weeks post-intervention and percentage change. However, in the pre-menopausal population, no significant differences were observed in either between-group or within-group comparisons.

#### 3.3.2. Facial Bone Density

The results of facial bone density are shown in Table 5A,B and Figure 4C,D. When only post-menopausal participants were included in the analysis, the test food group showed significantly higher HU values than the placebo food group at 24 weeks post-intervention in the following facial bones: the cortical bone in the mandible 7-6, cortical and cancellous bone in the mandible 3-4, cortical bone in the suborbital foramen, and cortical bone in the mental area. Additionally, the change and percent change in HU values were significantly greater in the test food group than in the placebo food group at all sites analyzed in the facial bones.

When only pre-menopausal participants were included in the analysis, the test food group showed significantly higher HU values than the placebo food group at 24 weeks post-intervention in the following facial bones: the cortical bone in the mandible 1-1 and cancellous bone in the supraorbital foramen. Additionally, the change and percent change in HU values were significantly greater in the test food group than in the placebo food group at all sites analyzed in the facial bones.

#### 3.3.3. Bone Resorption Marker

The measured results of DPD, a bone resorption marker, are presented in Table 6. When considering only post-menopausal participants, DPD levels showed a significant increase in the placebo food group in both pre- and post-intervention measures in within-group comparisons. In the between-group comparisons, significantly lower DPD levels were identified in the test food group than in the placebo food group in pre- and post-intervention changes and percentage changes. However, in the pre-menopausal group, no significant differences were observed in either the within-group or between-group comparisons.

## 4. Discussion

This study found that continuous intake of calcium maltobionate resulted in significantly higher calcaneal bone density and lower levels of the bone resorption marker DPD compared to the placebo group, particularly in post-menopausal women. Moreover, calcium carbonate was unable to prevent the progression of deteriorated bone density in the facial bone, whereas the test food group, calcium maltobionate, could slow down the progress of deterioration and maintain it.

Previous studies have also shown the effectiveness of calcium maltobionate in reducing bone resorption in post-menopausal women [16]. However, while previous studies reported a decrease in DPD levels pre- and post-intervention with calcium maltobionate intake, in the present study, we observed a slight variation in DPD levels, indicating a different effect of calcium maltobionate intake on DPD. The previous studies were mainly crossover trials, whereas the present study is a parallel-group trial. The study also identified differences between groups in pre-intervention DPD values, which may contribute to the variation in DPD levels.

In this study, the bone mineral density of the calcaneus was evaluated using the QUS method, a method that involves bone density evaluation based on the transmission velocity of ultrasonic waves through the bone tissue. The QUS has been reported to strongly correlate with bone mineral content and bone density [26]. The QUS method has the benefit of being a radiation-free technique and can be performed routinely without limiting the number of exposures. The QUS method has also been correlated with the dual-energy X-ray absorptiometry (DEXA) method in assessing bone mineral density [27,28] and is one of the standardized and insured assessment methods in Japanese clinical practice [29,30]. The rate of change in YAM of calcaneal bone mineral density in the placebo group showed no significant changes in the pre-menopausal population (0%) compared with a reduction of 1.3% in the post-menopausal population, consistent with previous findings [31,32]. Comparison between groups revealed no statistical difference in calcaneal bone mineral density in the pre-menopausal population. In contrast, a significantly higher value was observed in the post-menopausal test group. This suggests a significant ameliorative effect of continuous calcium maltobionate intake on bone resorption progression (increased DPD level) and the decline in calcaneus bone density following menopause.

Bone mineral density assessment of the maxilla and other facial bones is challenging with DEXA and QUS methods; therefore, bone mineral density assessment using dental CT imaging was chosen. The HU value, used as a bone mineral density index by CT imaging, is strongly correlated with bone mineral density [33]. In clinical practice, dental CT photography devices are widely used for preoperative diagnosis of dental implant treatment, which provides a three-dimensional view of the bone and is widely used for the diagnosis of bone mass, bone quality, and bone and cartilage diseases [34,35]. The use of DEXA and CT techniques in assessing bone mineral density presents challenges [36,37]. In this study, the accuracy of fixed-point observations was enhanced by marking and positioning the analysis site using a surgical stent commonly used in implant procedure planning.

A decrease of approximately 1.5–3.5% was identified in facial bone mineral density in the placebo food group in both pre-menopausal and post-menopausal populations. Membranous bones, including the maxilla and other facial bones, formed by intramembranous ossification without cartilage precursors are more sensitive to bone loss than cartilaginous bones with cartilage precursors, with bone loss progressing from approximately age 40 years [11]. This study also revealed that the calcaneal bone density in the placebo food group was reduced more markedly around the mid-50 s after menopause (Figure 4B), whereas the trend (Figure 4D), in which facial bone density progressively decreased after age 40, irrespective of menopausal status, aligns with a previous study [11] suggesting a significant decrease in lumbar spine bone density after age 60, while facial bone density significant decreases after the age of 40–50. Post-intervention facial bone mineral density in the post-menopausal participants significantly increased in the test food group compared with the placebo food group, and as with DPD levels and calcaneal bone mineral density, a positive effect of calcium maltobionate intake was observed. Additionally, a significant increase in facial bone mineral density was observed in the pre-menopausal participants in the test food group, indicating a maintenance effect through continuous intake of calcium maltobionate. Therefore, assessing calcaneal bone mineral density and bone resorption markers, including DPD levels, may be helpful in the advanced stage of post-menopausal bone resorption in determining bone health. Furthermore, facial bone densitometry can be considered a valid evaluation index, including in men and pre-menopausal women, in whom increased bone resorption is still in its early stages.

For the prevention of osteoporosis, it is crucial to regularly monitor bone health consistently from an early stage before menopause and to review dietary and exercise habits [38]. Osteoporosis screening among Japanese individuals aged 40–70 years is very low at 4.5% [36]. Although bone mineral density assessment using dental CT is challenging, establishing a simpler method and diagnosing bone conditions in the pre-menopausal stage could prevent osteoporosis.

Reduced facial bone density, including in the mandible and maxilla, has been linked to increased wrinkles and decreased facial expressions due to the subsidence of the base and ridge of the skin covering the facial bone [39,40,41]. The decreased mandibular bone mass has also been associated with increases in the marionette line and maxilla, including the periorbital area, to the nasolabial groove [41,42,43]. This highlights the importance of maintaining bone density for cosmetic reasons, as changes in facial bone density affect apparent age. Raising awareness regarding the importance of maintaining bone density alongside skincare for appearance should increase the interest of youth in bone density, ultimately contributing to lower morbidity rates.

However, further studies are necessary to elucidate the mechanism by which calcium maltobionate intake improves facial bone density. This study has certain limitations. First, the clinical outcome for bone resorption was limited to DPD in this trial. DPD is one of the bridging components between type I collagen molecules, which is the main component of bone substrate, and it is excreted into urine as a result of bone resorption. However, it does not indicate bone absorption [44]. Further detailed assessment of the effect of calcium maltobionate intake on bone resorption requires a comprehensive analysis of the outcome, including TRACP-5b, a marker reflective of bone resorption activity. Second, due to the BDHQ used in the dietary survey, which is a questionnaire that collects information on the dietary habits followed in the previous month, the effect of calcium intake on bone density and the adverse effects of excessive intake may not have been accurately reflected. Although calcium intake before and during the intervention did not significantly differ between the groups, future studies should aim to comprehensively examine the effect of the test food on improving bone density and the adverse effects of excessive intake by including dietary surveys that record participants’ daily meals. Third, the test foods in this trial were restricted to tablets with a standardized formulation. Calcium absorption efficiency fluctuates in combination with various food components such as vitamin D, phosphorus, and oxalic acid [45]. Future research should investigate prescriptions containing calcium maltobionate for maximizing bone density improvements and combinations to be avoided.

## 5. Conclusions

An increase in bone resorption markers and a decrease in calcaneal bone mineral density were more pronounced in post-menopausal participants, whereas decreases in facial bone mineral density were observed in pre-menopausal participants. This suggests that facial bone mineral density could serve as a useful indicator for monitoring bone health from middle age onward. Furthermore, the continuous intake of calcium maltobionate appeared to mitigate bone density decline in both pre-menopausal and post-menopausal women, thus contributing to osteoporosis prevention.

## Figures and Tables

**Table 1:** Composition of test food and placebo food (daily doses).

	Test Food	Placebo Food
Glucose	3.434 g	3.434 g
Calcium maltobionate	1.100 g	―
Maltose	―	0.955 g
Calcium carbonate	―	0.145 g
Citric acid	0.182 g	0.182 g
Magnesium oxide	0.080 g	0.080 g
Ferric pyrophosphate	0.006 g	0.006 g
Silicon dioxide	0.052 g	0.052 g
Calcium stearate	0.103 g	0.103 g
Perfume	0.037 g	0.037 g
High-intensity sweetener	0.006 g	0.006 g
Energy	15.6 kcal	18.4 kcal
Carbohydrate	4.48 g	4.39 g
Protein	<0.005 g	<0.005 g
Fat	0.12 g	0.12 g
Calcium	73 mg	73 mg
Magnesium	48 mg	48 mg

**Table 2:** Characteristics of study participants.

Analysis Population			Test Food Group			Placebo Food Group		
Overall		*n*	24			24		
	Age		52.5	±	1.4	54.0	±	1.3
	Body height	Cm	156.9	±	0.9	159.2	±	5.6
	Body weight	Kg	50.6	±	1.1	53.6	±	1.4
	BMI	kg/m^2^	20.5	±	0.4	21.2	±	0.6
Post-menopausal		*N*	16			16		
	Age		56.2	±	1.0	56.9	±	1.0
	Body height	Cm	155.4	±	1.0	158.6	±	1.3
	Body weight	Kg	48.2	±	1.2	53.4	±	1.2
	BMI	kg/m^2^	20.0	±	0.5	21.3	±	0.5
Pre-menopausal		*N*	8			8		
	Age		45.1	±	2.0	48.3	±	2.1
	Body height	Cm	160.1	±	1.5	160.6	±	2.4
	Body weight	Kg	55.3	±	1.4	53.9	±	3.4
	BMI	kg/m^2^	21.6	±	0.5	21.0	±	1.5

Values are presented as the mean ± SE.

**Table 3:** Diet survey (brief-type diet history questionnaire).

Analysis Population	Item		Group	*n*	Pre-Ingestion			Post-24W		
Post-menopausal	Calories	kcal/day	Test food	16	1598	±	82	1717	±	87
			Placebo food	16	1658	±	63	1713	±	83
	Carbohydrate	g/day	Test food	16	192	±	11	216	±	16
			Placebo food	16	204	±	12	215	±	14
	Protein	g/day	Test food	16	68.9	±	5.5	66.3	±	5.4
			Placebo food	16	68.1	±	3.3	68.8	±	3.6
	Fat	g/day	Test food	16	55.9	±	4.0	54.3	±	3.2
			Placebo food	16	56.6	±	2.4	59.0	±	2.9
	Calcium	mg/day	Test food	16	617	±	32	613	±	52
			Placebo food	16	549	±	19	547	±	28
	Magnesium	mg/day	Test food	16	241	±	28	277	±	30
			Placebo food	16	250	±	26	262	±	24
	Phosphorus	mg/day	Test food	16	832	±	53	840	±	52
			Placebo food	16	867	±	51	848	±	51
	Potassium	mg/day	Test food	16	2732	±	175	2638	±	181
			Placebo food	16	2459	±	121	2476	±	144
	Vitamin D	µg/day	Test food	16	13.3	±	1.8	12.9	±	2.3
			Placebo food	16	13.1	±	1.4	12.2	±	1.4
	Vitamin K	µg/day	Test food	16	316	±	23	309	±	26
			Placebo food	16	296	±	22	314	±	26
Pre-menopausal	Calories	kcal/day	Test food	8	1690	±	133	1540	±	81
			Placebo food	8	1608	±	121	1635	±	86
	Carbohydrate	g/day	Test food	8	219	±	18	206	±	18
			Placebo food	8	198	±	16	189	±	13
	Protein	g/day	Test food	8	59.8	±	5.6	52.8	±	4.4
			Placebo food	8	63.3	±	7.5	69.0	±	9.0
	Fat	g/day	Test food	8	55.4	±	5.1	46.8	±	2.9
			Placebo food	8	53.1	±	7.3	54.2	±	6.2
	Calcium	mg/day	Test food	8	525	±	47	478	±	26
			Placebo food	8	549	±	47	570	±	49
	Magnesium	mg/day	Test food	8	293	±	29	301	±	22
			Placebo food	8	281	±	26	295	±	24
	Phosphorus	mg/day	Test food	8	855	±	50	879	±	55
			Placebo food	8	854	±	49	863	±	53
	Potassium	mg/day	Test food	8	2359	±	192	2287	±	147
			Placebo food	8	2891	±	314	2696	±	243
	Vitamin D	µg/day	Test food	8	8.9	±	0.8	8.2	±	1.2
			Placebo food	8	13.4	±	2.9	15.6	±	3.0
	Vitamin K	µg/day	Test food	8	254	±	26	246	±	48
			Placebo food	8	270	±	59	300	±	48

Post-24W: Post-24 week intervention. Values are presented as the mean ± SE.

**Table 4:** Changes in SOS and YAM values at the calcaneus.

Analysis Population			Group	*n*	Pre-Intervention			Post-24W				Pre-Intervention vs. Post-24W				Pre-Intervention vs. Post-24W			
												Amount of Change				Amount of Change (%)			
Post-menopausal	SOS	m/s	Test food	16	1521	±	12	1523	±	14		2.92	±	1.15	^#^ (*p* < 0.01)	0.19	±	0.49	
			Placebo food	16	1521	±	15	1519	±	14		−2.03	±	2.40		−0.13	±	0.11	
	YAM value	%	Test food	16	77.87	±	0.62	79.36	±	0.74	^#^ (*p* = 0.044)	1.49	±	0.40	^#^ (*p* < 0.01)	1.91	±	0.52	^#^ (*p* < 0.01)
			Placebo food	16	78.09	±	0.77	77.06	±	0.71		−1.04	±	0.36		−1.31	±	0.44	
Pre-menopausal	SOS	m/s	Test food	8	1529	±	20	1532	±	22		3.31	±	2.83		0.22	±	0.25	
			Placebo food	8	1529	±	20	1529	±	20		0.00	±	2.31		0.00	±	0.47	
	YAM value	%	Test food	8	82.04	±	1.07	83.73	±	1.19		1.69	±	0.83		2.08	±	1.04	
			Placebo food	8	82.04	±	1.07	82.04	±	1.08		0.00	±	0.31		0.00	±	0.37	

Values are presented as the mean ± SE. ^#^ *p* < 0.05 (vs. the placebo group). SOS: speed of sound, YAM: young adult mean, and Post-24W: post-24 week intervention.

**Table 5:** (**A**) Changes in Hounsfield unit (HU) values in the facial bone (cortical bone). (**B**) Changes in Hounsfield unit (HU) values in the facial bone (cancellous bone).

**A.**																			
Analysis population			Group	*n*	Pre-intervention			Post-24W				Pre-intervention vs. Post-24W				Pre-intervention vs. Post-24W			
												Amount of change				Amount of change (%)			
Post-menopausal	Mandible (7-6)	HU value	Test food	16	1173	±	15	1185	±	13	^#^ (*p* = 0.018)	11.2	±	4.4	^#^ (*p* < 0.01)	1.50	±	0.38	^#^ (*p* < 0.01)
			Placebo food	16	1165	±	12	1121	±	18		−44.1	±	8.2		−3.07	±	0.72	
	Mandible (4-3)	HU value	Test food	16	1053	±	15	1068	±	13		15.3	±	3.3	^#^ (*p* < 0.01)	1.49	±	0.31	^#^ (*p* < 0.01)
			Placebo food	16	1067	±	16	1032	±	15		−35.2	±	7.1		−3.25	±	0.65	
	Mandible (1-1)	HU value	Test food	16	927	±	14	940	±	13		13.8	±	2.9	^#^ (*p* < 0.01)	1.50	±	0.31	^#^ (*p* < 0.01)
			Placebo food	16	939	±	17	910	±	16		−29.2	±	6.8		−3.07	±	0.70	
	Mandible (3-4)	HU value	Test food	16	1074	±	16	1083	±	15	^#^ (*p* = 0.035)	8.2	±	5.1	^#^ (*p* < 0.01)	0.80	±	0.47	^#^ (*p* < 0.01)
			Placebo food	16	1060	±	17	1030	±	17		−30.1	±	6.9		−2.84	±	0.65	
	Mandible (6-7)	HU value	Test food	16	1184	±	20	1196	±	22		11.4	±	3.6	^#^ (*p* < 0.01)	0.93	±	0.30	^#^ (*p* < 0.01)
			Placebo food	16	1175	±	14	1134	±	15		−41.8	±	10.3		−3.52	±	0.86	
	Maxilla (supraorbital)	HU value	Test food	16	828	±	14	839	±	13		10.3	±	3.4	^#^ (*p* < 0.01)	1.30	±	0.43	^#^ (*p* < 0.01)
			Placebo food	16	829	±	14	802	±	15		−26.8	±	4.9		−3.26	±	0.60	
	Maxilla (suborbital)	HU value	Test food	16	978	±	13	1001	±	15	^#^ (*p* = 0.029)	22.8	±	5.6	^#^ (*p* < 0.01)	2.32	±	0.57	^#^ (*p* < 0.01)
			Placebo food	16	978	±	12	952	±	12		−25.9	±	4.7		−2.62	±	0.47	
	Mandible (mental)	HU value	Test food	16	954	±	9	966	±	10	^#^ (*p* = 0.020)	11.3	±	3.6	^#^ (*p* < 0.01)	1.17	±	0.38	^#^ (*p* < 0.01)
			Placebo food	16	957	±	9	929	±	10		−27.8	±	4.4		−2.91	±	0.47	
Pre-menopausal	Mandible (7-6)	HU value	Test food	8	1213	±	23	1228	±	21		15.4	±	7.3	^#^ (*p* < 0.01)	1.30	±	0.60	^#^ (*p* < 0.01)
			Placebo food	8	1230	±	22	1192	±	28		−38.5	±	16.2		−3.15	±	1.32	
	Mandible (4-3)	HU value	Test food	8	1155	±	24	1167	±	24		12.5	±	8.0	^#^ (*p* < 0.01)	1.10	±	0.70	^#^ (*p* < 0.01)
			Placebo food	8	1138	±	34	1104	±	34		−33.3	±	9.4		−2.95	±	0.85	
	Mandible (1-1)	HU value	Test food	8	1016	±	29	1051	±	29	^#^ (*p* = 0.041)	35.0	±	19.0	^#^ (*p* < 0.01)	3.58	±	1.96	^#^ (*p* < 0.01)
			Placebo food	8	968	±	38	935	±	38		−32.9	±	8.1		−3.40	±	0.88	
	Mandible (3-4)	HU value	Test food	8	1123	±	18	1142	±	20		18.3	±	5.4	^#^ (*p* < 0.01)	1.61	±	0.49	^#^ (*p* < 0.01)
			Placebo food	8	1130	±	28	1092	±	29		−38.5	±	11.9		−3.40	±	1.07	
	Mandible (6-7)	HU value	Test food	8	1233	±	34	1242	±	33		9.3	±	7.3	^#^ (*p* < 0.01)	0.79	±	0.60	^#^ (*p* < 0.01)
			Placebo food	8	1246	±	18	1217	±	23		−28.3	±	8.7		−2.30	±	0.72	
	Maxilla (supraorbital)	HU value	Test food	8	947	±	26	965	±	22		18.0	±	6.0	^#^ (*p* < 0.01)	2.01	±	0.69	^#^ (*p* < 0.01)
			Placebo food	8	936	±	25	916	±	24		−19.6	±	7.7		−2.06	±	0.84	
	Maxilla (suborbital)	HU value	Test food	8	1081	±	18	1101	±	17		20.7	±	6.4	^#^ (*p* < 0.01)	1.94	±	0.60	^#^ (*p* < 0.01)
			Placebo food	8	1063	±	22	1047	±	21		−16.0	±	8.6		−1.48	±	0.82	
	Mandible (mental)	HU value	Test food	8	1039	±	18	1050	±	18		10.8	±	5.1	^#^ (*p* < 0.01)	1.06	±	0.49	^#^ (*p* < 0.01)
			Placebo food	8	1027	±	18	1005	±	18		−22.1	±	8.5		−2.13	±	0.79	
**B.**																			
Analysis population			Group	*n*	Pre-intervention			Post-24W				Pre-intervention vs. Post-24W				Pre-intervention vs. Post-24W			
												Amount of change				Amount of change (%)			
Post-menopausal	Mandible (7-6)	HU value	Test food	16	251	±	6	254	±	6		2.5	±	1.0	^#^ (*p* < 0.01)	0.97	±	0.36	^#^ (*p* < 0.01)
			Placebo food	16	253	±	4	244	±	4		−9.2	±	2.2		−3.59	±	0.85	
	Mandible (4-3)	HU value	Test food	16	325	±	5	328	±	5		3.1	±	1.5	^#^ (*p* < 0.01)	0.98	±	0.45	^#^ (*p* < 0.01)
			Placebo food	16	322	±	6	312	±	7		−10.5	±	2.1		−3.26	±	0.64	
	Mandible (1-1)	HU value	Test food	16	459	±	8	465	±	7		6.3	±	1.2	^#^ (*p* < 0.01)	1.40	±	0.29	^#^ (*p* < 0.01)
			Placebo food	16	462	±	8	444	±	7		−17.5	±	3.0		−3.74	±	0.64	
	Mandible (3-4)	HU value	Test food	16	331	±	5	333	±	5	^#^ (*p* = 0.028)	1.5	±	1.7	^#^ (*p* < 0.01)	0.50	±	0.50	^#^ (*p* < 0.01)
			Placebo food	16	324	±	7	312	±	6		−12.1	±	2.3		−3.70	±	0.70	
	Mandible (6-7)	HU value	Test food	16	245	±	6	247	±	7		2.2	±	1.6	^#^ (*p* < 0.01)	0.82	±	0.67	^#^ (*p* < 0.01)
			Placebo food	16	242	±	5	235	±	5		−7.0	±	1.9		−2.92	±	0.76	
	Maxilla (supraorbital)	HU value	Test food	16	347	±	6	350	±	6		2.8	±	1.5	^#^ (*p* < 0.01)	0.80	±	0.43	^#^ (*p* < 0.01)
			Placebo food	16	346	±	8	341	±	8		−4.9	±	1.4		−1.43	±	0.40	
	Maxilla (suborbital)	HU value	Test food	16	313	±	5	315	±	6		2.1	±	1.1	^#^ (*p* < 0.01)	0.66	±	0.35	^#^ (*p* < 0.01)
			Placebo food	16	315	±	7	307	±	7		−8.2	±	1.3		−2.66	±	0.44	
	Mandible (mental)	HU value	Test food	16	393	±	7	396	±	7		2.5	±	1.4	^#^ (*p* < 0.01)	0.64	±	0.35	^#^ (*p* < 0.01)
			Placebo food	16	390	±	8	384	±	7		−6.6	±	1.4		−1.68	±	0.36	
Pre-menopausal	Mandible (7-6)	HU value	Test food	8	260	±	10	263	±	10		3.1	±	1.1	^#^ (*p* < 0.01)	1.13	±	0.37	^#^ (*p* < 0.01)
			Placebo food	8	270	±	11	261	±	11		−9.9	±	2.1		−3.75	±	0.89	
	Mandible (4-3)	HU value	Test food	8	357	±	10	360	±	10		3.3	±	2.6	^#^ (*p* < 0.01)	0.98	±	0.72	^#^ (*p* < 0.01)
			Placebo food	8	347	±	10	335	±	11		−12.4	±	3.8		−3.60	±	1.09	
	Mandible (1-1)	HU value	Test food	8	513	±	10	507	±	11		−5.6	±	1.9	^#^ (*p* < 0.01)	−1.11	±	0.37	^#^ (*p* < 0.01)
			Placebo food	8	510	±	10	494	±	10		−16.7	±	5.2		−3.23	±	1.00	
	Mandible (3-4)	HU value	Test food	8	356	±	8	360	±	8		3.9	±	2.5	^#^ (*p* < 0.01)	1.14	±	0.70	^#^ (*p* < 0.01)
			Placebo food	8	354	±	9	346	±	10		−7.8	±	2.9		−2.27	±	0.84	
	Mandible (6-7)	HU value	Test food	8	270	±	9	272	±	9		2.3	±	2.0	^#^ (*p* < 0.01)	0.85	±	0.71	^#^ (*p* < 0.01)
			Placebo food	8	277	±	8	271	±	9		−6.3	±	2.1		−2.37	±	0.87	
	Maxilla (supraorbital)	HU value	Test food	8	406	±	7	411	±	7	^#^ (*p* = 0.040)	5.0	±	2.1	^#^ (*p* < 0.01)	1.26	±	0.52	^#^ (*p* < 0.01)
			Placebo food	8	393	±	8	385	±	9		−7.3	±	2.2		−1.86	±	0.58	
	Maxilla (suborbital)	HU value	Test food	8	372	±	6	376	±	7		3.6	±	2.3	^#^ (*p* < 0.01)	0.96	±	0.63	^#^ (*p* < 0.01)
			Placebo food	8	362	±	8	355	±	7		−7.3	±	1.9		−2.00	±	0.54	
	Mandible (mental)	HU value	Test food	8	465	±	13	468	±	13		2.1	±	2.3	^#^ (*p* < 0.01)	0.44	±	0.48	^#^ (*p* < 0.01)
			Placebo food	8	442	±	16	433	±	16		−8.8	±	2.0		−2.00	±	0.43	

Values are presented as the mean ± SE. ^#^ *p* < 0.05 (vs. the placebo food group). Post-24W: post-24 week intervention.

**Table 6:** Changes in u-DPD (bone resorption markers).

Analysis Population		Group	*n*	Pre-Intervention			Post-24W				Pre-Intervention vs. Post-24W				Pre-Intervention vs. Post-24W			
											Amount of Change				Amount of Change (%)			
Post-menopausal	nmol/mmol·Cr	Test food	16	5.13	±	0.31	5.32	±	0.40		0.20	±	0.27	^#^ (*p* = 0.047)	3.82	±	5.23	^#^ (*p* < 0.01)
		Placebo food	16	4.53	±	0.23	5.50	±	0.34	* (*p* = 0.024)	0.97	±	0.33		24.32	±	8.47	
Pre-menopausal	nmol/mmol·Cr	Test food	8	4.31	±	0.16	4.08	±	0.23		−0.23	±	0.30		−4.31	±	7.02	
		Placebo food	8	4.50	±	0.38	5.04	±	0.32		0.54	±	0.39		15.91	±	9.02	

Values are the mean ± SE. * p < 0.05 (vs. pre-intervention). ^#^ *p* < 0.05 (vs. the placebo group).

## Data Availability

The data presented in this study have been anonymized but include individual physical information and are available upon request from the corresponding author.
